# Urine Organic Acids as Potential Biomarkers for Autism-Spectrum Disorder in Chinese Children

**DOI:** 10.3389/fncel.2019.00150

**Published:** 2019-04-30

**Authors:** Qiao Chen, You Qiao, Xin-jie Xu, Xin You, Ying Tao

**Affiliations:** ^1^Department of Rheumatology and Clinical Immunology, Peking Union Medical College Hospital, Chinese Academy of Medical Sciences & Peking Union Medical College, Beijing, China; ^2^Central Research Laboratory, Department of Scientific Research, Peking Union Medical College Hospital, Chinese Academy of Medical Sciences and Peking Union Medical College, Beijing, China; ^3^Key Laboratory of Rheumatology and Clinical Immunology, Ministry of Education, Beijing, China; ^4^Autism Special Fund, Peking Union Medical Foundation, Beijing, China; ^5^Institute of Artificial Intelligence, Ping An Technology (Shenzhen) Ltd., Beijing, China

**Keywords:** autism spectrum disorder, biomarker, urine organic acids, Chinese, metabolomics, diagnosis

## Abstract

Autism spectrum disorder (ASD) is a neurodevelopmental disorder that lacks clear biological biomarkers. Existing diagnostic methods focus on behavioral and performance characteristics, which complicates the diagnosis of patients younger than 3 years-old. The purpose of this study is to characterize metabolic features of ASD that could be used to identify potential biomarkers for diagnosis and exploration of ASD etiology. We used gas chromatography-mass spectrometry (GC/MS) to evaluate major metabolic fluctuations in 76 organic acids present in urine from 156 children with ASD and from 64 non-autistic children. Three algorithms, Partial Least Squares-Discriminant Analysis (PLS-DA), Support Vector Machine (SVM), and eXtreme Gradient Boosting (XGBoost), were used to develop models to distinguish ASD from typically developing (TD) children and to detect potential biomarkers. In an independent testing set, full model of XGBoost with all 76 acids achieved an AUR of 0.94, while reduced model with top 20 acids discovered by voting from these three algorithms achieved 0.93 and represent a good collection of potential ASD biomarkers. In summary, urine organic acids detection with GC/MS combined with XGBoost algorithm could represent a novel and accurate strategy for diagnosis of autism and the discovered potential biomarkers could be valuable for future research on the pathogenesis of autism and possible interventions.

## Introduction

Autism spectrum disorder (ASD) is a developmental disorder characterized by impaired communication and social behavior, as well as displays of restricted and repetitive behavior (Keller and Persico, 2003). Although the pathogenesis of autism is uncertain, it is considered to involve an interaction between multiple genetic and environmental risk factors that are present in the few first years of life (Nair, 2000).

The diagnostic criteria for ASD require that symptoms become apparent in early childhood, typically before age three (Dieme et al., 2015). Autism diagnosis currently relies on scales and professional surveyors using behavioral methods. For instance, the Diagnostic and Statistical Manual of Mental Disorders, Fourth Edition (DSM-4) is the 1994 version of the American Psychiatric Association (APA) that provides a classification and diagnostic tool for ASD. Early identification and early intervention of autistic children are recognized as two of the most crucial factors for improving outcomes for individuals affected by ASD (Dawson et al., 2010, 2012; Warren et al., 2011; Zwaigenbaum et al., 2013; Klin et al., 2015). However, due to the challenges of early ASD diagnosis many older children miss the best intervention period.

Metabolic abnormalities associated with ASD include: phenylketonuria (PKU), disorders in purine metabolism, folate deficiency in brain development, succinic semialdehyde dehydrogenase deficiency, Smith-Lemli-Opitz syndrome (SLOS), organic acidurias (e.g., pyridoxine dependency, 3-methylcrotonyl-CoA carboxylase deficiency, and propionic acidemia), and mitochondrial disorders (Manzi et al., 2008; Zecavati and Spence, 2009; Ghaziuddin and Alowain, 2013). The presence of psychiatric, behavioral, and developmental regression together with metabolic disorders in autism (Wanders et al., 1999; Wang et al., 1999; Cox et al., 2001; Kompare and Rizzo, 2008) requires studies concerning the relationship between these pathological states and whether metabolic products of amino acid and lipid synthesis in urine or blood could be autism biomarkers (Schain and Freedman, 1961; Hanley et al., 1977; Bull et al., 2003; Kałużna-Czaplińska, 2011). After glomerular filtration and tubular condensation, the macromolecular proteins in the blood can be filtered and the urine becomes a concentrated organic acid. The natural physiological role of the kidney makes urine the best specimen for analyzing organic acid metabolism.

Several previous studies focused on organic acid biomarkers in autistic patients. Emond et al. (2013) found that levels of citrate, succinate, and glycolate were significantly increased in the urine sample of ASD children, whereas Mavel et al. (2013) found that β-alanine, glycine, taurine, and succinic acid levels were increased in the urine sample. Another study indicated that around 10 metabolites significantly differed between an autism group and the control group (Kałużna-Czaplińska, 2011). Some organic acids were highlighted by multiple studies, while others were seen only in a specific study. In general, microbial metabolites, niacin metabolism, mitochondria-related metabolites, and amino acid metabolites are the most common perturbations in autistic children. These results illustrates the complexity of metabolic disorders and etiology in autistic patients, leading to the exploration of building models for multivariate analysis. A metabolomics study of urine in 22 ASD children and 24 controls built an orthogonal partial least-squares discriminant analysis (OPLS-DA) model (AUROC = 0.91) (Hu, 2003), another one based on 14 ASD and 10 controls obtained a Principal Component Analysis (PCA) model (AUROC = 0.775) and identified a set of organic acids as potential biomarkers (Kałużna-Czaplińska, 2011). These studies do have some limitations, such as different races, limited regions, single algorithm used, and limited sample size. Similar researches with large sample size on Chinese children have rarely been reported. Moreover, some recently developed machine learning algorithms, such as XGBoost, have shown better performance over traditional algorithms on many tasks beyond biomedical domain. Therefore, we launched this representative study of a Chinese population with a larger sample size and a few more recent algorithms.

The aims of this study were to identify metabolic signatures of ASD and to find potential biomarkers for autism diagnosis and possible etiology. We used gas chromatography-mass spectrometry (GC/MS) to assess major metabolic perturbations in organic acid levels in urine from children with autism versus non-autistic subjects. Considering the complexity of ASD, the rise or fall of different organic acids is insufficient. A model using classification analyses of collected data for multiple organic acids that exhibit significant differences between healthy and autistic individuals should be feasible and may allow autistic patients to be distinguished.

## Materials and Methods

### Participants

This prospective study involved children who had autism (AU) and typically developing children (TD) over the period from December 2014 through May 2018. Children in the autistic group were enrolled from the Beijing Herun Clinic. This study was approved by the Peking Union Medical College Hospital (study #ZS-824), written informed consent was obtained from the parents of the participants. All participants were examined by experienced pediatricians.

Inclusion criteria for Autistic Disorder (AU) were as defined by the Diagnostic and Statistical Manual of Mental Disorders, Fourth Edition (DSM-4) (Hu, 2003). All autistic children were assessed by specialized clinicians.

Exclusion criteria were: (1) presence of other diseases such as diabetes or PKU; (2) presence of certain factors that would interfere with the detection of urine organic acids (e.g., renal failure, hepatic insufficiency, dietary intervention therapy); (3) diagnosis of other neuropsychiatric disorders; (4) parents who could not complete the assessment.

Typically developing children were enrolled from primary schools in Beijing.

### Procedures

Several precautions were strictly followed both before and after sampling to ensure specimen quality. The precautions and sampling steps were:

Before sampling:

(1) The subjects could not have used antibiotics (oral or infusion) in the previous month. Since some indicators we detect are associated with the intestinal micro-environment, antibiotic usage could affect the results by altering the distribution of intestinal flora.
(2) Both groups were not allowed to take probiotics within 2 weeks of sample collection. Probiotics can also perturb the intestinal micro-environment and affect the accuracy of urine organic acid testing.
(3) Study participants were not allowed to consume fruits or tomatoes within 24 h of sample collection due to the phenol or acid contents of these foods. For example, apples contain polyphenols such as anthocyanins, flavanols, phenolic acids, and catechins. Grapes are also rich in polyphenols. Such compounds can affect various metabolic pathways, and could affect the consistency of our results.

#### Sampling Steps

Midstream urine from the first morning void was collected in sterile tubes. The samples were placed on dry ice or in a freezer as soon as possible to avoid bacterial growth.

### Measures

Information concerning study population characteristics was obtained from the Peking Union Medical College Hospital electronic medical record system. Follow-up information was collected through regular clinic and telephone communication.

All assessments of the children’s behavior and dietary habits were provided by the parents and professional third-party institutions. The forms were produced in strict accordance with relevant standards and were completed following delivery of a detailed introduction and description of the study. Samples were collected either in the home or outpatient environment to ensure that external factors did not affect the samples. The researcher confirmed by phone that study guidelines were being followed.

The urine samples were assayed at the Great Plains Laboratory, Inc. (Lenexa, KS, United States). The GC/MS was performed as described in a previous research (Shaw et al., 1995). Due to the limitation of available data, only concentrations of 76 organic acids were reported from the spectrum analysis. Before analyses, all sample concentrations were normalized with urine creatinine as a way of minimizing variability due to differences in urine concentration.

### Data Processing and Modeling

**FIGURE 1:**
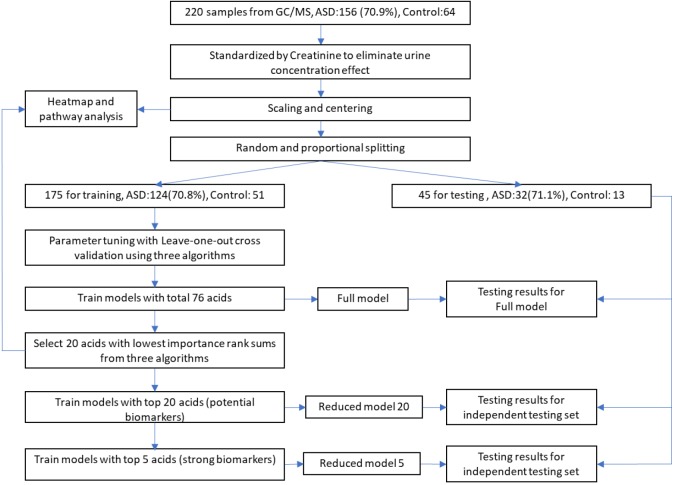
Workflow of data processing and modeling. After standardization, scaling, and centering, sample data were split into training and testing sets, while testing set would be strictly excluded from any model building processes to minimize overfitting. Potential biomarkers were discovered by selecting top N acids from an importance rank sum list generated by three different classification algorithms, while N was determined from testing results of different *N*-values. The discovered biomarkers’ potential mechanisms were investigated through heatmap along with associated metabolic pathways.

The total processes of data processing and modeling are illustrated in Figure 1. Sample data from GC/MS were first standardized by creatinine to eliminate urine concentration variabilities. Then the data were further processed with scaling and centering. To avoid data contamination between model building and model testing processes, we set aside an independent testing set from the entire data set in advance. The independent testing set would be strictly excluded from any model building processes so that overfitting effect in testing stage could be minimized. The splitting between testing set and training set were through a random process, while the ratios of control and ASD samples were kept approximately equal in these two sets.

During the model building process, we first trained our models and adjusted algorithm parameters using the training set with all 76 organic acids by maximizing AURs from leave-one-out cross validations. The modeling algorithms included Partial Least Squares Discriminant Analysis (PLS-DA, R mixOmics package), Support Vector Machine (SVM, R e1071 package), and XGBoost (eXtreme Gradient Boosting, R XGBoost package). The generated models with total 76 acids were designated as full models. Then, full models’ classification performance was evaluated using the independent testing set.

To identify potential biomarkers for ASD, we exploited a voting mechanism from all three algorithms to avoid possible biases. First, importance scores of all acids were determined by all the three algorithms using R caret package (Gevrey et al., 2003). Next, each algorithm provided a rank order of all acids according to their importance scores. Then, a list of acids with each acid’s sum of importance rank from the three algorithms in descending order was generated. Last, we trained models with only top N acids on the list and tested their classification performance on the testing set to identify the possible biomarkers. The models with top a few acids are referred as reduced models. Biomarkers were determined by observing the testing results of these reduced models on the testing set.

After the detection of biomarkers, to investigate possible mechanism behind them, we produced a heatmap with hierarchical clusters of all sample data and mark these biomarkers on the map after standardization processes. The heatmap was split aligning two dimensions, sample groups, and related metabolic pathways.

Classification algorithms were evaluated using AURs and their confidence intervals were estimated using bootstrapping methods with 2,000 bootstrap steps. Mann–Whitney *U*-test was used to compare the values for important biomarker acids. Multiple comparisons were adjusted using the false discovery rate (FDR) method (Benjamini and Yekutieli, 2001). Part of evaluations of PLS-DA algorithm was conducted using SIMCA-P Version 11.5 (Umetrics, Umeå, Sweden).

## Results

A total of 220 participants were enrolled including 156 autism patients (ASD group) and 64 typically developing children (TD group). The population characteristics of the ASD and TD groups have been summarized in Table 1. Among the ASD group, 80.13% were male. In TD group, 73.44% were male. The ASD and TD groups showed no significant differences in gender (*P* = 0.285).

**Table 1:** Characteristics of ASD group and TD group.

	ASD	TD
	*n* = 156	*n* = 64
Age, years	6 (4, 9.75)^∗^	5 (4, 7)^∗^
No. of males (%)	125 (80.13%)	47 (73.44%)
No. of females (%)	31 (19.87%)	17 (26.56%)

*All values are expressed as numbers (percentages) or median value (P25, P75). A total of 220 participants (156 autism patients and 64 typically developing children) were enrolled and divided into the ASD group and TD group. Among the ASD group, 80.13% were male. In TD group, 73.44% were male. The ASD and TD groups showed no significant differences in gender (P = 0.285). ^∗^median values (P25, P75).*

### Data Sets

Two sets, a training set (80%) and a testing set (20%), were randomly separated from the total data set, and each had a similar proportion of ASD children. The training set contained 124 (70.9%) ASD children and 51 TD children, whereas the testing set had 32 (71.1%) ASD and 13 TD children.

### Model Building Using Training Set

Using training set, we fine-tuned parameters of the three algorithms. For PLS-DA, the best major parameter, Ncomp, is 2. For SVM, we obtained the best results using linear kernel. For XGBoost, we optimized three major parameters, max_depth, eta, and nrounds with optimal values of 2, 0.15, and 200, respectively.

The model building process employed leave-one-out cross validation as guidance for parameter tuning. The final results of the full models in this process were shown in Table 2. The AURs for these three algorithms were 0.864 (PLS-DA), 0.833 (SVM), and 0.931 (XGBoost) in training set with leave-one-out cross validation.

**Table 2:** Potential marker metabolites found in GC/MS of urine samples.

No	Metabolite	Differentiation for autistic samples	*p*-value^∗^ after FDR adjustment
1	Phenylactic	↑	0.000
2	3-Hydroxy-3-methylglutaric	↑	0.004
3	Phosphoric	↓	0.001
4	Fumaric	↓	0.003
5	3-Oxoglutaric	↓	0.001
6	Aconitic	↓	0.000
7	*N*-Acetylcysteine (NAC)	↓	0.056
8	Malonic	↓	0.031
9	Tricarballylic	↓	0.052
10	Glycolic	↓	0.140
11	Creatinine	↑	0.010
12	Malic	↓	0.055
13	Oxalic	↑	0.025
14	Tartaric	↓	0.046
15	Pyruvic	↑	0.013
16	4-Cresol	↑	0.030
17	Carboxycitric	↓	0.001
18	3-Hydroxyglutaric	↓	0.071
19	2-Hydroxybutyric	↑	0.330
20	2-Oxoglutaric	↓	0.408

*A voting mechanism from all three algorithms was applied to generate the most important 20 organic acids as potential biomarkers for ASD. We performed U-Mann–Whitney test to determine whether there were significant differences in the levels of these 20 organic acids between ASD and TD children. ^∗^p-values were calculated using Mann–Whitney test; ↑, increased level compared with TD; ↓, decreased level compared with TD.*

**FIGURE 2:**
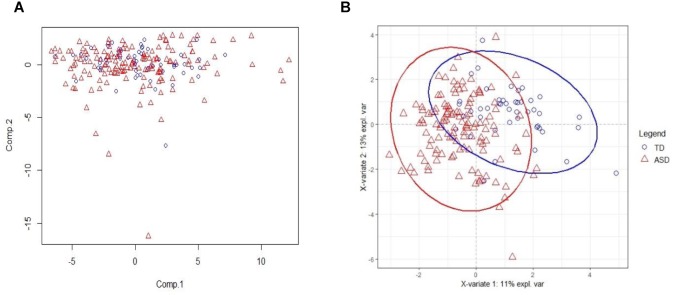
PCA and PLSDA score plots. **(A)** The Principal Component Analysis (PCA) score plot on training set with all 75 acids. **(B)** The PLS-DA score plot on training set with selected 20 biomarker acids. With first two components, R2X (cum) = 0.26, R2Y (cum) = 0.535, Q2 (cum) = 0.386.

The PCA result on training set with all acids is on Figure 2A. From the figure, we see that PCA could not distinguish between ASD and TD groups, since the new components variables generated with maximal variances might not be aligned with the outcome groups. However, it does identify some outliers. To make models more robust, we did not remove these outliers in the following analyses.

### Model Testing Using Independent Testing Set

To avoid any possible overfitting, we tested the full model on the independent testing set and obtained AURs of 0.863 (PLS-DA), 0.791 (SVM), and 0.94 (XGBoost). These results had shown similar values with those in training stage showing that training stage has generated little overfitting to the training set.

### Potential Marker Metabolites

**FIGURE 3:**
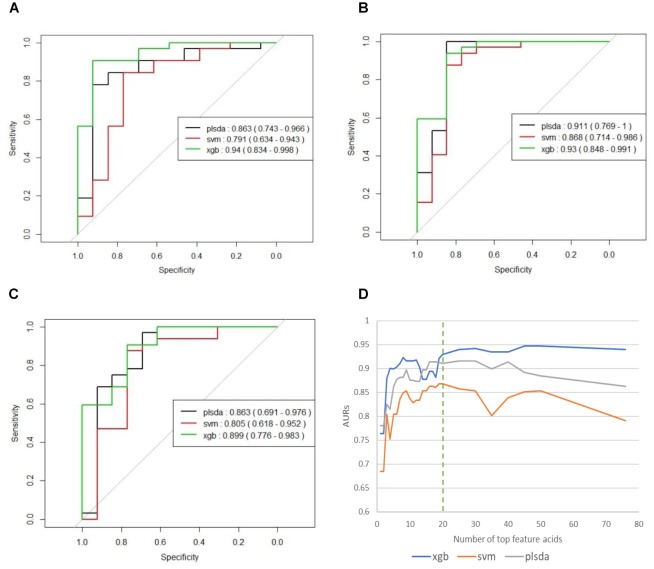
**(A)** ROCs of final models on independent testing set based on all 76 organic acids. **(B)** ROCs of final models on independent testing set based on top 20 organic acids. **(C)** ROCs of final models on independent testing set based on top five organic acids. **(D)** Curves of AURs against selected top acids. Top 20 acids represent the best collection of possible ASD biomarkers, while adding more will make AURs for SVM and PLS-DA decrease and make AUR for XGB appear platformed.

We used the testing resulting of reduced models to identify potential markers. The results of these reduced models are shown in Supplementary Table S1 and Figure 3. Figure 3D the curves of AURs against different N selected top acids on testing set. Clearly, top 20 acids represent the best collection of possible ASD biomarkers, while adding more acids to the model will make AURs for SVM and PLS-DA decrease and make AUR for XGB appear platformed (The ensemble mechanism of XGBoost might make it more robust to irrelevant features). Actually, XGBoost achieved an AUR of 0.93 which was very close to the value of 0.94 in full model, and this suggest that these top 20 acids could capture most of the features of ASD. Even top 5 acids could get an AUR of 0.899.

The 20 identified potential marker metabolites are listed in Table 2. Their levels compared with TD group are also shown in Table 2. Using these 20 identified marker metabolites, we draw the score plot of PLA-DA of training set on Figure 2B. There is a separation between TD and ASD groups with R2X (cum) = 0.26, R2Y (cum) = 0.535, Q2 (cum) = 0.386, *p*-value of CV-anova = 1.26183e-006.

### Heatmap Analysis of Metabolic Pathway

We tried to use heatmap with hierarchical clustering to discovery possible related metabolic pathways (Supplementary Figure S1). Rows of the heatmap represent different samples from TD and ASD groups, while columns represent different metabolites grouped in different metabolic pathways. The pathway names are listed in the figure legend. The heatmap shows that the identified biomarker metabolite acids are distributed across a wide variety of pathways: Intestinal Microbial Overgrowth, Amino Acid Metabolism, nutritional, Krebs Cycle, Oxalate Metabolism, Glycolytic Cycle, and Mineral Metabolism. This diverse distribution suggests that these organic acids may act on a variety of metabolic pathways and reflects the complexity of metabolic abnormalities in autism.

## Discussion

To identify metabolic signatures of ASD and find organic acids in urine that could act as potential biomarkers for diagnosis and disease treatment, three algorithms (PLS-DA, SVM, and XGBoost) were used to analyze GC/MS data for urine samples. The results showed the effectiveness of this method in distinguishing ASD children from TD children. XGBoost model produced the best results (AUROC = 0.94) among the three algorithms. The modeling was performed on the basis of all 76 organic acids, among which the top 20 acids were identified as potential biomarkers with a voting mechanism from all three algorithms. To go a step further, we selected top 5 acids as strong biomarkers. The amount of phenylactic acid was significantly higher in the ASD group, whereas the amounts of aconitic acid, phosphoric acid, 3-oxoglutaric acid, and carboxycitric acid were significantly lower in the ASD group. These organic acids are involved in a variety of metabolic pathways including amino acid metabolism, intestinal flora, energy metabolism (Krebs Cycle), and bone salt metabolism. Although a total of 76 organic acids contributed to modeling, we just involved the top 5 ones in discussion part since they made significant contributions in modeling, which may indicate the major metabolic abnormality of autism.

### Complex Relationships Among Urinary Organic Acids and ASD Pathogenesis

The heatmap generated from GC/MS analysis of urinary organic acids showed the complex relationship among these compounds in ASD. Several organic acids were in the same pathway, whereas others are involved in multiple pathways. To date, the metabolites that have been explored as possible ASD biomarkers include: nutritional markers, microbiome metabolites, amino acid metabolites, Krebs cycle metabolites, pyrimidine metabolites, neurotransmitter metabolites, products of ketone, and fatty acid oxidation and mineral metabolism, as well as indicators of detoxification and fluid intake (e.g., creatinine) (Kałużna-Czaplińska, 2011).

These organic acids may affect the function of intestinal flora. In our study, we also collected stool specimens from the study participants. Analysis of stool samples and the intestinal flora is underway, and the abundance of intestinal flora combined with findings for urinary organic acid metabolism should strengthen the diagnostic potential of these compounds.

The organic acids we identified may affect nervous system development and thus we included assessments of neurological symptoms (e.g., unexplained excitability or mania) on the study scales. We will examine whether the severity of these symptoms in ASD and the CARS and ABC scores are relevant in a future study.

### The Diagnostic Potential of the Established Model

Calibration and optimization of parameters is a critical step in model building. Three algorithms (PLS-DA, SVM, and XGBoost) were examined to achieve this task. Among them, the first two algorithms, PLS-DA and SVM, were previously described (West et al., 2014; Dieme et al., 2015). To our knowledge, application of the XGBoost algorithm in a model of urine organic acids to distinguish children with ASD from TD children has not been previously reported.

Among the three algorithms, XGBoost had an AOC of 0.94. Use of the XGBoost algorithm is an innovation in autism-related research (Chen and Guestrin, 2016), and the efficiency of this model differs from that described in earlier studies. XGBoost has been proved to have better performance than other more traditional models in many machine learning tasks outside biomedical domains. This is largely due to its built-in ensemble mechanism and its ability to capture non-linear features. In contrast, traditional linear algorithm for metabolite analysis, PLS-DA, is limited in capturing non-linear relations. This has also been observed in this study. In addition, XGBoost also shows more robustness to adding more irrelevant features than SVM and PLA-DA. Conclusively, the establishment of this model increases the possibility of early diagnosis of autism. The examination of organic acids in urine is non-invasive and relatively inexpensive, the requirements for sample collection are not strict and the operability is very high.

### Notable Changes in Urinary Organic Acid Levels in ASD Patients

The PLS-DA score plot shows a clear distinction among the distribution of metabolite profiles between TD and ASD children. Our analyses showed that 5 urinary organic acids had significant differences between ASD and TD children and thus could have diagnostic potential as ASD biomarkers.

The ASD group had higher levels of phenylactic acid but decreased amounts of aconitic acid, phosphoric acid, 3-oxoglutaric acid, and carboxycitric acid compared to TD children. These metabolites are associated with multiple biochemical processes (Koulman et al., 2009). Phenylactic acid is a byproduct of amino acid metabolism, and the higher levels seen for ASD children could indicate abnormalities in the function of enzymes involved in amino acid metabolism. Moreover, phenylactic acid can inhibit the growth of Gram-negative and Gram-positive bacteria, as well as some fungi. Thus, elevated phenylactic acid levels could inhibit the normal function of the intestinal microflora and exacerbate metabolic disorders. Intestinal microbes can affect neurotransmitter production in the central nervous system and in turn affect the induction of endogenous sensations, production of bacterial metabolites, and mucosal immune-related activity (Carabotti et al., 2015). Moreover, phenylactic acid is a metabolite of phenethylamine, which acts as a monoaminergic neuromodulator and as a neurotransmitter in the human central nervous system to promote neuron excitation (Sabelli et al., 1976).

Also in the context of intestinal flora, levels of carboxycitric acid and 3-oxoglutarate acid were significantly decreased in the ASD group relative to the TD group. Carboxycitric acid can be a marker of intestinal microbial overgrowth, particularly yeast and fungi. Certain strains of the mold *Aspergillus niger* have efficient citric acid production and can be used for industrial-scale citric acid production (Lotfy et al., 2007). Although to our knowledge, this study is the first to report a significant decrease of carboxycitric acid in urine samples from ASD children, other studies identified intestinal microbe metabolites as potential agents that can affect nervous system function. Meanwhile, carboxycitric acid, a product of the Krebs Cycle, showed decreased levels in our assays and may be indicative of energy metabolism disorders in children with autism. We also found that 3-oxoglutarate, a common metabolite of yeast and fungi (Thomas et al., 2010; MacFabe et al., 2011; Kocovska et al., 2012), was significantly lower in children with autism. The low concentrations of both carboxycitric acid and 3-oxoglutarate that we observed in urine from autistic patients could be due to increased uptake of these compounds across the blood-brain barrier of the brain. Our results are consistent with previous studies that showed anti-fungal treatments for children with autism can effectively reduce the amounts of corresponding organic acid indicators (Cobb and Cobb, 2010), and suggests that gastrointestinal yeast could provide a basis for dietary adjustments such as gluten/casein-free diets that are important for children’s nervous system development and could mitigate autism symptoms. 3-oxoglutarate in urine is associated with the presence of harmful gut flora such as *Candida albicans* (Schmidt, 1994). These results support the reliability of the gut-brain axis and suggest new avenues of study for autism.

Aconitic acid is produced from citric acid dehydration that occurs during the Krebs Cycle and is a marker of mitochondrial activity. Mitochondrial disease, either through maternal inheritance or other causes, is present in up to 5% of autistic children (Rossignol and Frye, 2012; Frye et al., 2013). Previous studies reported that *cis*-acotinic acid levels are increased in children with autism (Noto et al., 2014; Mussap et al., 2016). Here, we found that acotinic acid levels were decreased in the ASD group relative to the TD group, which is indicative of energy metabolism deficiencies in energy metabolism of ASD. In the Krebs Cycle, citrate undergoes stereospecific isomerization to isocitrate by the enzyme aconitase hydratase and the intermediate *cis*-aconitate (Mussap et al., 2016). Meanwhile, *trans*-aconitic acid (TAA) acts as an anti-inflammatory agent in plant-based treatments for rheumatoid arthritis used in Brazil, and could be one explanation for the decreased levels of aconitic acid in the ASD group. Similarly, it has been reported that inflammatory mediators may play crucial role in some neuropsychiatric diseases. Dan et al. (2015) found that homocysteine (Hcy) and uric acid (UA) may contribute to the pathogenesis of multiple system atrophy (MSA) and serum Hcy together with UA levels could be a diagnostic tool of MSA (AUROC = 0.736). In addition, another cross-sectional study supported that low serum UA levels may indicate a higher risk of Parkinson’s disease (PD) and serum UA level could serve as an indirect biomarker of prediction in PD (Mengqiu et al., 2013).

Phosphoric acid is important for bone metabolism. In our study population we observed decreased amounts of phosphoric acid in ASD children relative to TD children, which could suggest that ASD pathology involves abnormal bone metabolism, although this possibility requires further investigation. Vitamin D regulates bone formation and density by promoting absorption of key intestinal compounds such as calcium and phosphate. Imbalances in phosphoric acid could be related to an imbalance of several other substances. In pregnant women, vitamin D deficiencies can affect regulatory T cell function and in turn immune responses. Such vitamin D deficiencies can impact the developing fetus and could increase the risk for autism. Vitamin D is also critical during development of the fetal nervous system through regulation of the expression of several nerve growth factors as well as transforming factor beta 2 (TGF-b2) and neurotrophin 3 and 4. Previous studies showed that some children with autism have vitamin D deficiency (Pioggia et al., 2014; Uğur and Gürkan, 2014). The amount of serum 25 (OH) D_3_ is significantly lower in children with ASD, indicating that lower 25 (OH) D levels could be an independent risk factor for autism, and may be independently associated with disease severity (Gong et al., 2014). Our findings support observations of disorders in bone salt metabolism in children with autism, and are also consistent with clinical symptoms indicating that reduced bone mineral density is common in children with autism.

## Conclusion

In this study, we used GC/MS to evaluate major metabolic fluctuations in 76 organic acids present in urine from 156 children with ASD and from 64 non-autistic children. Three algorithms, Partial Least Squares-Discriminant Analysis (PLS-DA), Support Vector Machine (SVM), and eXtreme Gradient Boosting (XGBoost), were used to develop models to distinguish ASD from TD children and to detect potential biomarkers. By a voting mechanism, 20 acids have been successfully identified as potential ASD biomarkers and reduced model with top 20 acids achieved 0.93 and represent a good collection of potential ASD biomarkers. These biomarkers were distributed across a wide variety of metabolic pathways, indicating the complicated mechanism behind ASD. XGBoost algorithm has shown better classification performance and more robustness than other traditional algorithms.

In summary, urine organic acids detection with GC/MS combined with XGBoost algorithm could represent a novel, non-invasive and accurate strategy for diagnosis of autism and the discovered potential biomarkers could be valuable for future research on the pathogenesis of autism and possible interventions, and have a range of clinical applications.

## Ethics Statement

This study was carried out in accordance with the recommendations of Chinese Academy of Medical Sciences Peking Union Medical College Hospital Ethics Review Committee The protocol was approved by the Chinese Academy of Medical Sciences Peking Union Medical College Hospital Ethics Review Committee.

## Author Contributions

QC, YQ, XY, and X-jX contributed to the conception and design of the study. XY and X-jX enrolled the patients and controls, and collected all the clinical data, and urine samples. QC and YQ transferred the data to a database, analyzed the data, and wrote the first draft of the manuscript. YT analyzed the data, set up diagnosis model, and revised the figures. All authors contributed to the manuscript revision, and read, and approved the submitted version of the manuscript.

## Conflict of Interest Statement

YT was employed by Ping An Technology (Shenzhen) Ltd., Institute of Artificial Intelligence, Beijing, China. The remaining authors declare that the research was conducted in the absence of any commercial or financial relationships that could be construed as a potential conflict of interest.
