# Corrigendum: Urine Organic Acids as Potential Biomarkers for Autism-Spectrum Disorder in Chinese Children

**DOI:** 10.3389/fncel.2019.00388

**Published:** 2019-08-22

**Authors:** Qiao Chen, You Qiao, Xin-jie Xu, Xin You, Ying Tao

**Affiliations:** ^1^Department of Rheumatology and Clinical Immunology, Peking Union Medical College Hospital, Chinese Academy of Medical Sciences & Peking Union Medical College, Beijing, China; ^2^Central Research Laboratory, Department of Scientific Research, Peking Union Medical College Hospital, Chinese Academy of Medical Sciences and Peking Union Medical College, Beijing, China; ^3^Key Laboratory of Rheumatology and Clinical Immunology, Ministry of Education, Beijing, China; ^4^Autism Special Fund, Peking Union Medical Foundation, Beijing, China; ^5^Institute of Artificial Intelligence, Ping an Technology (Shenzhen) Ltd., Beijing, China

**Keywords:** autism spectrum disorder, biomarker, urine organic acids, Chinese, metabolomics, diagnosis

In the original article, there is an error in the corresponding author order. The first corresponding author should be “Xin You” followed by “Ying Tao”.

The authors apologize for this error and state that this does not change the scientific conclusions of the article in any way. The original article has been updated.

