# Supplementary figures and images for: Urine Organic Acids as Potential Biomarkers for Autism-Spectrum Disorder in Chinese Children
Source: Front Cell Neurosci. 2019 Apr 30;13:150. doi: 10.3389/fncel.2019.00150 (PMC6502994; doi:10.3389/fncel.2019.00150)

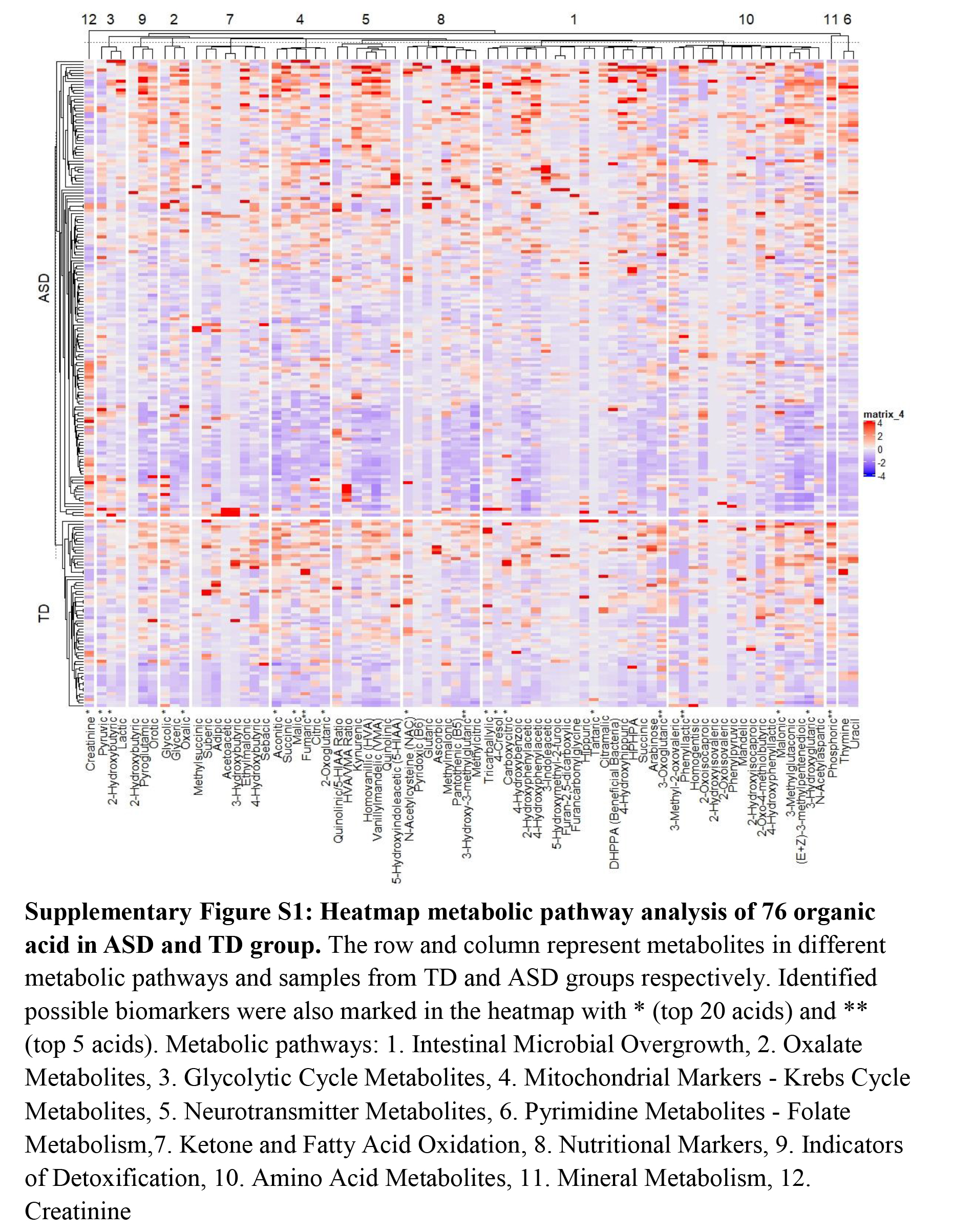

Supplement: Supplementary file 1 [file Image_1.JPEG]
