# Supplementary material for: Urine Organic Acids as Potential Biomarkers for Autism-Spectrum Disorder in Chinese Children
Source: Front Cell Neurosci. 2019 Apr 30;13:150. doi: 10.3389/fncel.2019.00150 (PMC6502994; doi:10.3389/fncel.2019.00150)
Supplement: Supplementary file 2 [file Table_1.docx]

**Supplementary Table S1.** Evaluation results of final models on two data sets ^#^

|  | Training set | | Testing set | |
| --- | --- | --- | --- | --- |
|  | ASD (n=124) | Control (n=51) | ASD (n=32) | Control (n=13) |
| **PLS-DA** (Ncomp=2) |  | |  | |
| Full model | 0.864 (0.808-0.916) * | | 0.863 (0.743-0.966) | |
| Reduced model of top 20 | 0.859 (0.804-0.918) | | 0.911 (0.762-1) | |
| Reduced model of top 5 | 0.807 (0.725-0.883) | | 0.863 (0.687-0.978) | |
| **SVM** (kernel='linear') |  | |  | |
| Full model | 0.833 (0.758-0.9) | | 0.791 (0.634-0.943) | |
| Reduced model of top 20 | 0.868 (0.798-0.917) | | 0.868 (0.714-0.99) | |
| Reduced model of top 5 | 0.763 (0.686-0.824) | | 0.805 (0.613-0.938) | |
| **XGBoost** (max_depth = 2, eta =0.15, nrounds =200) |  | |  | |
| Full model | **0.931** (0.889-0.963) | | **0.940** (0.834-0.998) | |
| Reduced model of top 20 | **0.937** (0.9-0.97) | | **0.930** (0.831-1) | |
| Reduced model of top 5 | **0.914** (0.869-0.957) | | **0.899** ((0.774-0.986) | |

#: Evaluation results are represented using AURs. Results on training set were from leave-one-out cross validation. *：95% confidence intervals were estimated by Bootstrap for 2000 times.

The Confidence intervals were estimated by Bootstrapp for 2000 times. The AUROC for the three algorithms on the training set were 0.864 (PLS-DA), 0.833 (SVM) and 0.931 (XGBoost), and the corresponding AUROC were 0.863 (PLS-DA), 0.791 (SVM) and 0.94 (XGBoost) for the testing set, which are shown in ROC curves.
